# Extirpation of an Osseous Tumor of the Upper Jaw
*Dr. Whitehead sends us full particulars of this interesting case, (which was first published in the *medical Record*), with the result of treatment up to December, 21st, 1869.—Ed.


**Published:** 1870-02

**Authors:** W. R. Whitehead

**Affiliations:** (Univ. of Paris).


### ARTICLE III.

### *Extirpation of an Osseous Tumor of the Upper Jaw.**

By W. R. Whitehead, M. D. (Univ. of Paris).

Osseous growths of the jaws present many points of interest, which are revealed by a careful study of their anatomical appearances and clinical features. It is a subject eminently worthy of remark, and might be appropriately considered at this time. I have, however, on a previous occasion endeavored to describe succinctly this variety of diseased growth of the upper jaw, in connection with other tumours of this region equally interesting and deserving of notice.† More than a general notice of the subject now would be departing from the simple design and original object of this communication; which is the description of a case of excision of the superior maxilla, of peculiar importance, and attended with the most favorable results.

---

* Dr. Whitehead sends us full particulars of this interesting case, (which was first published in the *Medical Record*), with the result of treatment up to December, 21st, 1869.—Ed.

†See Remarks on certain tumors of the Superior maxilla. New York Medical Journal. Vol. III. 1866. No. XVII. And also in No XV. of the same volume the report of a case of excision of this bone for an osseous tumor.

Case.—Miss F. P——, from the South, aged eighteen, of good constitution, well formed, and slightly above the medium size, was afflicted with a tumor of the upper jaw. She came to New York on the 28th of December, 1866, and placed herself under my care for the purpose of having it removed. A few weeks prior to this date, I was consulted by her, at which time I examined the growth, and became acquainted with its previous history. At three years of age her nurse let her fall, and she received a blow, which bruised her right cheek; when six years old, a slight enlargement of the cheek on that side was first observed, which, however, was not very apparent, and caused her parents no uneasiness; but at the age of ten, the enlargement had sufficiently increased to excite their apprehensions.

Many medical gentlemen were consulted, who differed widely in their opinions regarding its nature. Topical remedies were ineffectually used, and constitutional means unavailingly essayed. About four years ago, the parents of the young lady visited Richmond with her, and consulted a distinguished surgeon, the late Dr. Chas. Bell Gibson, who advised the excision of the tumor. This gentleman expressed an unwillingness to give chloroform in the operation, and terrible apprehensions of pain deterred the parties from permitting it to be undertaken without the use of an anæsthetic. She suffered constantly from headache, during a year preceding the removal of the tumour. Two or three months previous to its excision, it grew rapidly, especially towards the orbit, and the headache increased with its extension in this direction. Last fall she had chills and fevers, which persisted irregularly until within a few weeks of the day of the operation. My first examination of the patient permitted me to observe that there was considerable deformity of the face, caused by a tumor occupying the right superior maxillary bone, slightly distorting the mouth, and making the cheek quite tense. This tumor invaded the orbit, and pressing upwards, narrowed the orbital cavity in its transverse direction, and compressed the eye. The roof of the mouth of the right side was slightly depressed. By passing the end of my index-finger behind the extremity of the alveolar arch, feeling and circumscribing the posterior and external surfaces of the tumor as thorougly as possible, and carefully observing the deformity caused by it, I could judge with a certain degree of precision the extent of the disease. The mass appeared ovoid, had a smooth and regular surface and to every part accessible to the touch was exceedingly hard on pressure, which was without pain.

The headache, from which she had so long suffered, appeared to be due to compression of the second branch of the trifacial nerve, and its anastomosing branches. The two last molar teeth were gone, and the remaining molar tooth was much decayed; the other teeth on that side were sound. The voice was but slightly nasal or indistinct. Mastication was not impaired to any extent, and the patient swallowed without difficulty. Apprehensions of the continued increase of the tumor, and its rapid extension recently towards the brain, excited the fears of the young lady, and caused her to desire the removal of the growth without unnecessary delay; and although its extirpation was the principal and most urgent reason for the operation, yet a very natural solicitude about the extent of deformity which might remain afterwards, was to her a source of considerable disquietude, and made me desirous to prevent, if possible, cicatricial marks about the face, resulting from incisions of the cheek or other part conspicuously apparent.

Believing that moderate sized tumors of the upper jaw could be removed without cutting the lip or cheek—an opinion which has already been expressed by Ferguson and some others, though not convincingly demonstrated—I experimented on the cadaver with a view to determine the degree of mobility of the parts about the mouth, and also the extent to which this cavity can be stretched by the use of well adapted means. These experiments were simple, and appeared satisfactory. They consisted in exposing thoroughly the bony opening of the nares, by a dissection of the soft parts from the anterior nasal spine of the maxillæ, and thus facilitating the dissection of the anterior surface of either of these bones, as high up as the orbit, and rendering the nasal and malar processes quite accessible to bone-scissors and other instruments. By the use of cheek retractors, a gradual and very considerable dilatation of the mouth was effected. These experiments encouraged me to hope that I might be able to remove the tumor without cutting the lip or cheek of the patient. The only objection which occurred to me was, that the distension of the cheek or distortion of the parts might, if the mass were larger than previous examination had enabled me to determine, render the anterior part of the tumor as high up as the orbit, inaccessible to dissection. Being prepared, however, as well as possible against all contingencies, and provided with suitable instruments, efficiently assisted, and ready to avail myself of any advantages which could not be anticipated, on the second of January 1867, I proceeded to remove the diseased growth, and to test the value of the experiments previously made on the cadaver.

*Operation.*—The patient was seated in a dentist's chair procured for the occasion ; and after she had taken a small quantity of brandy and water, ether was administered to her, and the operation commenced by detaching, with a pair of strong scissors, the soft parts from the anterior spine of the maxillæ, and partially exposing to view the anterior bony opening of the nares. At this time the patient commenced to vomit; she had, however, not taken any breakfast that morning, and the cause of the vomiting was attributed to the ether. Considerable delay was caused by this interruption of the operation. Ether was not again used, but after she had swallowed more brandy and water, she was rapidly made insensible to pain with chloroform, and my dissection of the gum from the tumor continued. The cheek retractors provided for forcibly stretching the orifice of the mouth were well applied, and produced a proper extent of dilatation, which could not have been much increased. I

soon became convinced that the tumor in this case was too large and distended the cheek too much to be removed without an external incision. My previous opinion, predicated on the facility with which I could manipulate the parts on the cadaver, was modified by the peculiar extension upwards of this growth, which made its anterior and upper surfaces less accessible to instruments than could be anticipated before the preliminary incisions of the gum. Reserving, therefore, this operative procedure for a case more favorable to its adoption, I promptly cut through the middle of the lip in its furrow, and extended the incision around and behind the right nostril, a short distance up the side of the nose. This incision, which is recommended by Ferguson, permitted me to complete the operation rapidly and with facility. The nasal branch of the maxilla was cut with Liston's bone-scissors, and the malar bone effectively severed with a metacarpal saw. An incision of the soft parts was then made along the posterior border of the palate bone, from the end of the alveolar arch to the middle palatine suture, and another at right angles to this incision, terminating at the alveolar of the left incisor, which was extracted. One blade of Liston's scissors was passed into the right opening of the bony nares, and the other into the mouth, and the palatine arch cut. The tumor was then wrenched out with a pair of Ferguson's lion forceps, and the remaining soft parts adherent to it were detached. One of the little palatine arteries, throwing out a fine jet of blood, was secured, after a little trouble, by a ligature ; with this exception there was no trouble from hæmorrhage, but to guard against it considerable lint, some of which was saturated with a solution of Squibb's sub-sulphate of iron, was stuffed into the cavity left after the extirpation of the tumor.

The upper two-thirds of the external incision were united with ten or twelve silver wire sutures, and the cut surfaces of the lip were held in apposition with two figure-of-eight sutures. With the valuable assistance of Dr. Nathan Bozeman, now of this city, but late of Montgomery, Ala., this external incision was very neatly, accurately, and securely approximated throughout its entire length. After the application of a light dressing to the part, the patient was conveyed to her bed, and half a grain of sulphate of morphia prescribed, to be taken at night. In a few hours the pulse commenced to rise, and in the night she had considerable fever.

*January* 3.—Early in the morning I found the pulse 120; strong and full. She could swallow without difficulty; took strong beef-tea every few hours, and her mouth was often mopped out with a weak solution of chlorinated soda, and occasionally with glycerine. She took a few times during the day an anodyne and diaphoretic mixture, containing sulphate of morphia and swt. spts. of nitre; had very little thirst; she became restless, and late in the night her pulse increased to about 130.

*January* 4.—I saw her early in the morning, and commenced to give her stimulants; very soon after swallowing about half an ounce of sherry wine, her pulse was lowered eight or ten beats to the minute. She took frequently during the day brandy-whey, and beef essence; and eight drops of muriated tincture of iron in solution were administered to her every few hours. In the middle of the day her pulse was 120 to 135, and about the same at my evening visit. The same mouth-washes were used as at last visit.

*January* 5.—She continued to take the beef essence and tincture of iron as before; but substituted sherry wine for brandy-whey. By means of a rubber syringe with a long pipe, the upper part of her mouth was washed out several times with tepid suds of Castile soap; afterwards with clear water, and then with water containing a drop or two of creasote. In the evening she was quite restless, had a hot dry skin, and her pulse was increased a few beats.

*January* 6.—I saw her at an early hour and found her much better; her pulse was only 96 to 100. Last night she was very uneasy and restless up to twelve o'clock; about that time she got rid of a lint plug which was annoying her; but soon afterwards she had a profuse perspiration, and improved rapidly. She told the attendants that she was glad that I would find her in the morning so much better. She complained of hunger, and drank her beef tea very gratefully. To-day the hare-lip pins were removed, and the union of the lip was found to be perfect. The same detersive and medicated injections were used as yesterday. She took some chicken broth, and continued to take the beef essence and tincture of iron. A gentle aperient having failed to move her bowels, they were slightly relieved by a suitable clyster.

*January* 7.—Rested well last night; this morning a piece of lint became partially detached and caused her some discomfort; I removed this piece, and one or two others, with a pair of dressing forceps; syringed out the mouth with Castile suds, clear water, and weak creasote water. Continued the same medicines and nourishment. At the evening visit I removed two or three pieces of lint.

*January* 8.—Took out all the silver sutures; washed the parts and put fresh isinglass to the lip and side of the nose, and removed all the lint plugs. I entertained doubts, as I had a few days before, whether the offensive suppuration caused by the lint, was fully compensated by the supposed security afforded against hæmorrhage by cramming the wound with this material. If all other necessary means for stopping hæmorrhage are made available, and the parts exposed sufficiently long to the air, I have no doubt that sometimes no lint would be required. It would be far better to have only the saliva, which is bland and unirritating, in contact with the surfaces of the wound, than lint. In a similar operation, about two years ago, which was attended with most excellent results, I abstained from stuffing lint into the cavity which remained after the excision of the tumor; and I believe that it would be well, if possible, always to adhere to this practice.

To-day she is able to sit up and syringe out her mouth herself; has very little fever; said at the morning visit, that she felt quite comfortable. Although I have requested her to observe strict silence, she will occasionally address a sprightly remark to her sister, or utter some pleasant expression, indicative of her habitually cheerful disposition.

*January* 9.—She continues to improve; has a relish for food; took some soft boiled eggs this morning; uses the same mouth washes; discontinued the tincture of iron.

*January* 10.—Last night she was restless, and about eleven o'clock she had considerable fever; I was told to-day that once or twice before she has had fever during the night Fifteen grains of sulphate of quinia were prescribed to be given in clyster, but only a third of it at a time, and at three hours' interval.

*January* 11.—Doing very well; took in the same way as yesterday, twelve grains of quinine.

*January* 12.—She has not had a return of her fever; but the quinine was repeated once more.

*January* 18.—During the last six or eight days, she has been able to sit up and to walk about the house; and, if the weather were pleasant, could go out to walk. She went out this morning, in a closed carriage, and had her photograph taken, which compared with the one taken before the operation, exhibits a marked improvement in her personal appearance, effected by the operation that relieved her of this encroaching tumor. Desirous of having the deficiency of the upper-jaw, caused by excision of the tumor, repaired by some competent dentist, I made careful inquiry, and learned through a professional friend, that in a similar operation performed in 1842 by Dr. R. D. Mussy, of Cincinnati, on the patient Thomas McGilligan, Dr. H. Crane, dentist of this city, made a substitute for the upper maxillary bone which the patient had worn twelve years when last seen. The contrivance which the dentist Cook made, and to which Mussy refers in his publication of the case, was soon rejected by the patient as useless. Miss P. visited to-day the rooms of Dr. Crane, who is an ingenious and skillful dentist. With a little effort she can articulate quite distinctly; this is a very considerable advantage, and will aid in obtaining an excellent result, when this gentleman makes for her an artificial palate and set of teeth.

*January* 23.—She left to-day for her home. About two weeks after the operation, she was well enough to leave, but was detained by snow storms, which interrupted railway travel. I have recently received a letter from her, dated February 6th, and will quote a few lines from it :

"I am improving very rapidly, my color has returned with my strength, and I am sure I have already gained several pounds in weight; the soreness has almost entirely left my mouth, and it is healing up very nicely."

*April* 3.—Miss P. has returned to New York to place herself under the care of the dentist. The parts are entirely healed ; she is no longer subject to headache, and is in the most excellent health and spirits. The space left after the extirpation of the tumor, has closed up remarkably well ; the entire soft palate is preserved ; and the only opening in the roof of the mouth is an oval orifice about an inch and a quarter in its longest diameter, which is antero-posterior. The eye is well retained in its natural position, and its movements are free, and easily executed. There is some flatness of the cheek, and distortion of the right side of the mouth, which is obviously caused by the disuse af the muscles on that side of the face, which defect can probably be removed by the device of the dentist.

*April* 18.—Dr. Crane has been eminently succesful in accurately fitting to the young lady's mouth an ingenious piece of workmanship, composed in part of vulcanite, with a beautiful arch of teeth, and rose colored gums of porcelain, appearing so natural that they defy close scrutiny. This dental attachment, which she has worn several days, enables her to pronounce with perfect distinctness ; it improves her appearance, and her mouth feels more comfortable with it than without it, as it causes not the slightest irritation or inconvenience ; indeed it is a most remarkable triumph of the dental art, and has, I believe never been excelled. Having accomplished the object of her last visit to New York, she is ready to return to her home, and now enjoys social intercourse with a zest which she rarely experienced before the removal of the tumor.

*December* 21st, 1869.—Patient in excellent health a few weeks ago—new plate adjusted to mouth by dentist—(Dr. C.)

*Examination of the Tumor.*—The tumor, after its excision, was heavy and hard, and appeared like a compact mass of hypertrophied bone, weighing four ounces and a quarter, and involves the superior maxilla and part of the malar bone. The greatest thickness of this growth is measured by its vertical diameter; its superior surface corresponds to the floor of the orbit, which before the operation, in being displaced upwards, diminished the orbital cavity in this direction to the extent of about half an inch. The inner fractured border of this surface corresponds to the lower border of the orbital plate of the ethmoid bone, from which the tumor was separated in wrenching it out. On the upper third of its anterior surface may be seen an indentation, corresponding to the external orifice of the suborbital canal, from which point may be measured with the eye the extent of encroachment of the tumor upwards. Its external surface projects quite prominently, and its inferior and posterior surfaces also exhibit considerable enlargement of the maxillary bone. A piece of the tumor was sliced out by sawing to its centre; and the internal structure of the mass was observed to be similar to that of the most compact bone tumor, and which is known as the ivory exostosis. Exceedingly fine sections of this piece were placed on slips of glass and successively examined, under the microscope, after adding a drop of glycerine to each delicate section, and pressing gently its thin glass cover. Numerous osseous lacunæ with their diverging canaliculi, were seen thickly studding the little masses within the field of the instrument. These osseous lacunæ were found to be present in each specimen examined, though not so numerous in some as in others.

The tumor seems to be formed exclusively of bone tissue, and may be considered as a type of dense osseous tumors of the superior maxilla, and belongs to an interesting variety of benign growths.

*Conclusions.*—The growth is of a benign character. The origin of the diseased action in the bone is traceable to a contusion.

The upward extension of the tumor was peculiarly unfavorable; the mass invaded slowly the orbital cavity, and probably would ultimately have caused a protrusion of the eye and an encroachment on the brain.

My experience in this operation has been entirely favorable to the use of chloroform. As an excellent precautionary measure, it is well to give brandy in this as in all surgical operations, before administering chloroform; the brandy facilitates the action of the chloroform, and materially diminishes the chances of accident resulting from the depressing effects of this agent on the nervous system.

I believe that tumors of the upper jaw of a moderate size, when favorably situated, may be excised without an external incision of the face.

The incision in the furrow of the lip and up the side of the nose, offers peculiar advantages over other incisions of the face.

The structure of the tumor will influence the amount of hæmorrhage; causing it to be more or less dangerous and difficult to control, in proportion to the vascularity of the growth.

It is best, if possible, not to stuff lint into the cavity left after the extirpation of the tumor; leaving the parts to the bland and unirritating contact of the saliva.

An excellent substitute of vulcanite and porcelain may be made to repair the deficiency caused by excision of the upper maxillary bone; end thus the skill of the dentist may essentially aid in correcting the contraction of the parts after healing.

A removal of the entire floor of the orbit does not necessarily cause a falling of the eye; such was not the case after this operation.

